# Surgical treatment of thymic epithelial tumor and myasthenia gravis

**DOI:** 10.3389/fsurg.2024.1467789

**Published:** 2024-11-18

**Authors:** Gizem Özçıbık Işık, Akif Turna

**Affiliations:** Department of Thoracic Surgery, Istanbul University-Cerrahpaşa, Cerrahpaşa Medical Faculty, Istanbul, Türkiye

**Keywords:** thymic epithelial tumor, myasthenia gravis, thoracic surgery, minimal invasive surgery, thymoma, thymic carcinoma, thymectomy

## Abstract

Thymic epithelial tumors originate from the epithelial cells of the thymus and are typically diagnosed during the 5th and 6th decades of life. The incidence is consistent between men and women, averaging 1.7 cases per year. Thymomas, neuroendocrine tumors, and thymic carcinomas are subtypes of thymic epithelial tumors, with thymomas being the most prevalent (75%–80%) and thymic carcinomas following at 15%–20%. Thymoma and thymic carcinoma exhibit distinct disease courses; thymomas grow slowly and are confined to the thymus, while thymic carcinomas demonstrate rapid growth and metastasis. Overall survival rates vary, with a 78% 5-year survival rate for thymoma and a 30% rate for thymic carcinoma. Thymic epithelial tumors may be linked to paraneoplastic autoimmune diseases, including myasthenia gravis, hypogammaglobulinemia, pure red cell aplasia, Cushing's syndrome, systemic lupus erythematosus, and polymyositis. Staging of thymic epithelial tumors can be done according to Masaoka-Koga and/or TNM 8th staging systems. The treatment algorithm is primarily determined by resectability, with surgery (Extended Thymectomy) serving as the foundational treatment for early-stage patients (TNM stage I-IIIA, Masaoka-Koga stage I-III). Adjuvant radiotherapy or chemotherapy may be considered following surgery. In advanced or metastatic cases, chemotherapy is the first-line treatment, followed by surgery and radiotherapy for local control. Myasthenia gravis, an autoimmune disease presents with progressive muscle fatigue and diplopia. Positive antibodies (Anti-AChR, Anti-MuSK, LRP4) and electromyography aid in diagnosis, and approximately 10% of myasthenia gravis patients can also have thymoma. Treatment includes cholinesterase inhibitors and immunotherapy agents, with extended thymectomy serving as an effective surgical option for drug-resistant cases. Minimally invasive approaches (video-assisted thoracoscopic surgery or robot-assisted thoracoscopic surgery) have demonstrated comparable oncological outcomes to sternotomy, highlighting their effectiveness and reliability.

## 1 Introduction

### 1.1 Thymic epithelial tumors

A rare group of tumors known as thymic epithelial tumors (TETs) typically appears in the anterior mediastinum (1, 2). Epithelial cells and lymphocytes are the sources of thymic epithelial tumors (3). The average age of occurrence is similar in men and women, averaging 5th and 6th decades (3). Thymoma (Type A, AB, B1, B2, B3), thymic carcinoma (Type C), and thymic neuroendocrine tumor (NET) constitute subgroups of thymic epithelial tumors (2, 4, 5). The literature reports a thymoma incidence of 0.13–0.19 per 100,000 (4, 6, 7). Estimates place the incidence rate for neuroendocrine tumors at 0.02 per 100,000 and for thymic carcinomas at 0.07 per 100,000 (4, 7, 8). Although thymic epithelial tumors are rare, they are the most common tumor group in the anterior mediastinum (4, 6). The literature has not clarified the etiology, but it has not identified smoking, ionizing radiation, or alcohol as risk factors (9). The higher incidence of thymic epithelial tumors in Asians, African Americans, and Pacific Islanders supports the hypothesis of hereditary disease etiology (9). Some specific genomic changes have been detected in TETs; various chromosome deletions, translocations, and duplications can be observed according to subtypes (10).

Although they are usually clinically asymptomatic, paraneoplastic syndromes may occur in some patients (3, 11). Among the paraneoplastic autoimmune diseases associated with thymic epithelial tumors, hypogammaglobulinemia, pure red cell aplasia, Cushing's syndrome, systemic lupus erythematosus, and polymyositis, myasthenia gravis is the most common, although it occurs less frequently (11–13).

Thymomas have an overall 5-year survival of 90%, whereas distant metastases accompany thymic carcinomas, which have an overall 5-year survival of about 55% (9). TETs require long-term follow-up (e.g., 10 years) to evaluate overall survival and detect possible recurrences (9).

The 8th TNM staging is used in the anatomical staging of thymic epithelial tumors, but the 9th TNM criteria have also entered the literature (14–16). Table 1 displays TNM staging. In the 9th TNM staging, dividing the tumor size by 5 cm in the T evaluation is a newly added criterion (14, 16).Mediastinal pleural invasion was removed from the T classification and included as an additional histological factor (15, 16). Lung and phrenic nerve invasion were reduced to the T2 stage (14, 16). No changes were made in the N and M stages (14, 16) (Table 1).

**Table 1:** The 9th TNM staging criteria for thymic epithelial tumors.

*T*	Description		
T1	Tumor limited to thymus with or without encapsulation, or directly invades into to mediastinum alone, or directly invades the mediastinal pleura but does not involve any other mediastinal structure		
-T1a	≤ 5 cm		
-T1b	> 5 cm		
T2	Tumor directly invades the pericardium (either partial or full-thickness), the lung, the phrenic nerve		
T3	Tumor directly invades any of the following: - brachiocephalic vein, - superior vena cava, - chest wall, - extrapericardial pulmonary arteries or veins		
T4	Tumor directly invades any of the following: - aorta (ascending, arch, or descending), - arch vessels, - intrapericardial pulmonary artery or veins, - myocardium, - trachea, - esophagus		
*N*	Description		
N0	No nodal involvement		
N1	Anterior (Perithymic) nodes		
N2	Deep intrathoracic or cervical nodes (e.g., paratracheal, subcarinal, aortopulmonary window, hilar, jugular, and supraclavicular nodes)		
*M*	Description		
M0	No metastatic pleural, pericardial, or distant sites		
M1a	Separate pleural or pericardial nodule(s)		
M1b	Pulmonary intraparenchymal nodule or distant organ metastases		
Stage	*T*	*N*	*M*
I	T1a-b	N0	M0
II	T2	N0	M0
IIIA	T3	N0	M0
IIIB	T4	N0	M0
IVA	T any	N1	M0
	T any	N0,N1	M1a
IVB	T any	N2	M0, M1a
	T any	N any	M1b

Masaoka et al. staged thymic epithelial tumors based on capsule invasion and invasion into the surrounding fatty tissue (17). Koga and his colleagues also made significant contributions to the literature by developing the Masaoka-Koga staging, which focused on the invasion of surrounding tissues, lymphatic spread, and hematogenous spread (18, 19). Table 2 provides the Masaoka-Koga staging for thymic epithelial tumors (18, 19). In clinical use, TNM staging and Masaoka-Koga staging are used together (14, 19) (Tables 1, 2).

**Table 2:** The table shows the masaoka-koga staging for thymic epithelial tumors.

Stage	Definition
I	Grossly and microscopically completely encapsulated tumor
IIa	Microscopic transcapsular invasion
IIb	Macroscopic invasion into thymic or surrounding fatty tissue, or grossly adherent to but not breaking through mediastinal pleura or pericardium
III	Macroscopic invasion into neighboring organ (i.e., pericardium, great vessels, or lung)
IVa	Pleural or pericardial metastases
IVb	Lymphogenous or hematogenous metastases

Thymic epithelial tumor treatment varies according to subpathological type and stage (20). Clinical practice currently uses the 8th TNM in treatment options, but the increasing use of the 9th TNM will shift treatment to the 9th TNM in the coming years (15, 20, 21). The surgical margin negativity is the most important prognostic factor in the surgical treatment of thymic epithelial tumors (20, 21). For this reason, neoadjuvant treatments are used to achieve R0 resection (20, 22). Patients considered surgically inoperable benefit from the combination of chemotherapy and radiotherapy treatments, which also detect distant metastases (20, 22).

If myasthenia gravis is also present, the surgical treatment recommendation for thymoma, when evaluated according to subtypes, is total resection (extended thymectomy) (21, 23). Although minimally invasive approaches have advantages, open sternotomy remains the standard (21, 24).

Neoadjuvant radiotherapy and systemic treatment are not suitable for thymoma patients (21, 23). In TNM staging, the main treatment for the stage 1 thymoma group is surgery (21, 24). In medically inoperable stage 1 thymoma patients, chemotherapy or radiotherapy is recommended instead of chemoradiotherapy (21, 24). Extended thymectomy remains the gold standard treatment for stage 2 thymoma patients (21, 24). Despite the thought of neoadjuvant radiotherapy options being beneficial due to various invasions (pericardium, lung, phrenic nerve), we do not recommend neoadjuvant radiotherapy (21, 24). However, if R0 resection cannot be achieved, adjuvant radiotherapy options should be considered (21, 23). Systemic treatment is not recommended in stage 2 patients, and radiotherapy options are primarily recommended in medically inoperable patients (21, 23).

Surgery or direct surgery is the best way to treat stage 3 and stage 4 thymomas that can be removed after neoadjuvant treatment for R0 resection (21, 23). Neoadjuvant treatment is recommended as chemoradiotherapy or chemotherapy; in chemotherapy, cisplatin-based combination chemotherapy is recommended (21, 23). If it is possible to resect pleural and pericardial metastases in stage 4 thymoma, surgical treatment is recommended (21, 23). Open surgery (sternotomy) is superior to minimally invasive methods, and total resection is superior to partial resections (21). Unilateral phrenic nerve excision may be tolerated during surgery, but bilateral phrenic nerve excision is a contraindication (21). Adjuvant RT may be recommended if neoadjuvant RT has not been received, but there is no routine adjuvant RT recommendation (21, 23). In un-resectable stage 3 and 4 patients, radiotherapy and/or concurrent chemotherapy regimens are recommended (21, 23). In patients with recurrent thymoma, resection is recommended if there is a tumor in the thorax. Radiotherapy and platinum-based systemic chemotherapy treatments are parts of multimodal treatment (21, 23).

Patients with stage 1 and 2 thymic carcinoma should not receive neoadjuvant chemotherapy or radiotherapy; instead, they should undergo total resection with open surgery (21, 25). Adjuvant chemotherapy also has no place in treatment (21, 25). Radiotherapy is recommended for medically inoperable patients; chemotherapy has no place in the treatment (21, 25). It should not be forgotten that patients with stage 3 and 4 thymic carcinoma have locally advanced disease and multimodal treatment options (21, 25, 26). For R0 resection, surgical or direct surgical treatment options can be applied after neoadjuvant therapy (21, 26). Open surgery (sternotomy) is superior to minimally invasive methods, and total resection is superior to partial resections (21, 26). Unilateral phrenic nerve excision may be tolerated during surgery, but bilateral phrenic nerve excision is a contraindication (21). If neoadjuvant chemotherapy or radiotherapy is not given, they should be considered as adjuvant treatment options (21, 26). In un-resectable stage 3 and 4 patients, radiotherapy and/or concurrent chemotherapy regimens are recommended (21, 25, 26). When a tumor in the thorax is present in patients with recurrent thymic carcinoma, similar to thymoma, multimodal treatment includes radiotherapy and platinum-based systemic chemotherapy treatments (21, 25, 26).

Surgery is the first treatment option for stage 1–2 NETs, and R0 resection is the main treatment target (21, 27). If R0 resection cannot be achieved, radiotherapy and systemic chemotherapy (cisplatin + etoposide, carboplatin + etoposide) modalities should be added to the treatment (21, 27). For symptom control in Stage 3 and 4 NETs, consider adding local treatment options such as endobronchial therapy and ablation (21). Octreotide, lanreotide, everolimus, temozolomide ± capecitabine, or RT options are effective in low-grade typical carcinoids. In moderately atypical carcinoids, treatment options of cisplatin + etoposide, carboplatin + etoposide, temozolomide ± capecitabine, octreotide, lanreotide, and everolimus simultaneously with RT are effective (21).

In recent years, immunotherapy has emerged as a treatment option with increasing frequency. The literature reports a wide range of PD-L1 expression, including 25%–90% in thymomas and 40%–80% in thymic carcinomas (28–30). In this context, immune regulatory agents (Pembrolizumab and Avelumab) that block the PD-1/PD-L1 pathway may be a treatment option for patients with resistant thymoma and thymic carcinoma (28). However, immunotherapies are not standard treatments, and patients should exercise caution to avoid immune response-related side effects during treatment processes (28).

### 1.2 Myasthenia Gravis

Myasthenia gravis is a heterogeneous autoimmune disease (31). It is a neuromuscular junction disease that develops through antibody-dependent T lymphocytes (32). It has a general incidence of 0.3–2.8/100,000 years (33). Women between the ages of 20 and 30 are more likely to experience it, and it affects both men and women equally in middle and older ages (34). Approximately 10% of myasthenia gravis patients also have thymoma (33, 34). Myasthenia gravis manifests various clinical symptoms, with ptosis and diplopia being the most common ones (31, 35). Dysphagia, dyspnea, and extremity involvement are less common (31, 35). The clinical classification of myasthenia gravis uses the Osserman classification (31).

There are various clinical spectrums associated with myasthenia gravis, such as ocular involvement or generalized muscle involvement (31). The age of onset may be early or late (34, 36, 37). There may be single antibody positivity, multiple antibody positivity, or seronegativeness (36, 37) (Table 8). Patients with myasthenia gravis often exhibit various antibody positivities, with the most common being anti-acetylcholine receptor (Anti-AChR) positive, a condition where antibodies develop against the acetylcholine receptor (38–40). Less comm0only, patients may also exhibit antibodies against muscle-specific kinase (Anti-MuSK) and antibody positivity against lipoprotein receptor-related protein 4 (Anti-LRP4) (38–40).

Myasthenia gravis treatment varies according to clinical severity and classification (41). The most commonly used agent in the symptomatic treatment of mild myasthenia gravis is pyridostigmine, an acetylcholinesterase inhibitor (42). In moderate-to-severe myasthenia gravis, immunosuppressive drugs are added to the treatment (41, 42). Apply nonsteroidal immunosuppressive treatments when the daily dose of minimum effective prednisone exceeds 7.5 mg, as corticosteroid side effects are common (42). Azathioprine, tacrolimus, mycophenolate mofetil, methotrexate, and cyclosporine are among the nonsteroidal immunosuppressive agents used in the treatment of myasthenia gravis (42).

Antibodies activate the classical complement pathway (34). As a result of the systematically progressing complement system, C5b6789 (membrane attack complex (MAC)/terminal complement component (TCC)) is formed (34, 43). The membrane attack complex (MAC) destroys the postsynaptic membrane (34). The complement pathway's C5, C3, and C1 components are intermediate steps that enable the complement pathway to start and continue. Inhibitory agents for C5, C3, and C1 components provide improvement in clinical symptoms (34). Eculizumab, ravulizumab, and zilucoplan, developed against the C5 unit of complement, appear to be the most effective and successful agents in clinical practice (34, 43).

Developing B lymphocytes have the transmembrane protein CD20 on their surface, which regulates calcium flux (44). Progenitor cells and mature plasma cells do not express it (44). Rituximab is a monoclonal antibody that targets the CD20 protein, causing cell death upon binding to CD20 (44). When B lymphocytes die, the production of new antibodies ceases, leading to improvement in the myasthenia gravis clinic (44). Cell death occurs through antibody-dependent cellular cytotoxicity, complement-dependent cytotoxicity, the caspase pathway, and lysosomal activation (44).

Neonatal Fc receptor (FcRn) prolongs serum half-life by affecting immunoglobulin G (IgG) transport, distribution, and persistence (45). Traditional treatment methods for myasthenia gravis do not include FcRn inhibitors (45). However, the FcRn inhibitor efgartigimod has received FDA approval (45).

Drug-resistant cases can utilize the plasmapheresis method to stabilize the patient during the perioperative period (46). Agents such as intravenous immunoglobulin and subcutaneous immunoglobulin can aid in the maintenance treatment of myasthenia gravis (47). Aerobic exercises and respiratory muscle training are effective in treatment by increasing functional capacity (48) Thymectomy is among the effective treatment methods for patients with myasthenia gravis (49).

### 1.3 Surgical treatment

Thymus tissue originates embryologically from the third pharyngeal pouch and is anatomically located in the anterior mediastinum (50, 51). Thymus tissue consists of two lobes separated by septa and continues to grow until adolescence (50, 52). The inferior thyroid artery, the internal mammary artery, the internal thoracic artery, the pericardiophrenic artery, and the intercostal arteries all bring blood to the thymus (50). The counterparts of the arteries and the left innominate vein provide venous drainage (50). Lymphatic drainage occurs through parasternal, tracheobronchial, and brachiocephalic lymph nodes (50).

The thymus is a primary lymphoid organ that contributes to cellular immunity (50, 53). Thymus tissue consists of the cortex and medulla, with thymic lymphocytes located in the cortex (50, 54). Lymphocytes formed in the bone marrow come to the thymus tissue, where they undergo antigen-mediated induction and maturation of T lymphocytes into cytotoxic cells (50, 53).

Thymectomy indications are most commonly caused by thymoma and myasthenia gravis (50, 53). Thymectomy is also indicated in clinical conditions such as thymic carcinoma, neuroendocrine tumors, thymic cysts, and ectopic parathyroid glands located in the thymus (50, 55).

Before surgery, myasthenia gravis patients should receive clinical stabilization with cholinesterase inhibitors, intravenous immunoglobulin, and plasmapheresis agents to prevent myasthenic crises (50, 56, 57). Myasthenia gravis patients also need surgical anesthesia, and they should avoid using calcium channel blockers and magnesium (56). Excessive use of acetylcholinesterase inhibitors may lead to cholinergic crises (56, 57). The depressant effects of benzodiazepines and opiates on respiratory functions appear more clearly in myasthenia gravis patients (56). Myasthenia gravis patients exhibit resistance to depolarizing neuromuscular blocking agents (e.g., succinylcholine) and sensitivity to non-depolarizing neuromuscular blocking agents (e.g., rocuronium) due to the decrease in acetylcholine, indicating caution during the induction of anesthesia (56).

Patients with non-thymomatous anti-AChR-positive myasthenia gravis can benefit more after having a thymectomy (58–61).Wolfe and colleagues reported that, female gender, earlier onset of disease (<40 years of age) were associated with better outcome after thymectomy (58). Patients with myasthenia gravis over 50 years old who exhibit juvenile-onset or purely ocular symptoms were found to have limited treatment success with thymectomy (62). Extended thymectomy has been recommended in order to remove all of the thymus tissue in patients with myasthenia gravis or a thymic epithelial tumor (24, 60). An extended thymectomy may provide complete removal of the thymus tissue and the surrounding fatty tissue between the right and left phrenic nerves (63, 64). As with cancer, the tumor tissue must be completely removed and there must be no remaining thymus tissue to lower the rate of recurrence (24, 60).

Although sternotomy was initially the standard procedure for thymectomy surgery, technological advancements and the use of minimally invasive methods have demonstrated significant benefits in both the perioperative and postoperative course (50, 59). Minimally invasive methods consist of video-assisted thoracic surgery (VATS) and robotic-assisted thoracic surgery (RATS) (50, 59, 60). Even though minimally invasive methods stand out in terms of less pain, early discharge, cost, and aesthetics, they should not be preferred if oncological principles cannot be preserved (24, 50, 59, 65).

In general, contraindications to thymectomy include clinical conditions such as the patient's inability to tolerate general anesthesia, hemodynamic instability, and coagulopathy (50). However, once clinical improvement is achieved with medical treatment for the contraindication, thymectomy may be reconsidered (50). The complications of thymectomy can be listed as bleeding, pericardial injury, phrenic nerve damage, chylothorax, and pneumothorax (50, 65).

Myasthenia gravis is an autoimmune disease with different clinical spectrums (31, 34). Myasthenia gravis may also be associated with thymoma (35). Various antibodies, most commonly anti-acetylcholine receptor antibodies, may be present in patients with myasthenia gravis (38–40). Early-onset myasthenia gravis patients with anti acetylcholine antibody positivity are the most likely to benefit from surgery (58, 59). Extended thymectomy has been defined as the gold standard in myasthenia gravis surgery (63, 64). In an extended thymectomy, the thymus tissue between the right and left phrenic nerves and the fat tissue around them are completely cut out (63, 64). Applying simple thymectomy instead of extended thymectomy in myasthenia gravis patients results in recurrence after surgery (63, 64). Although simple thymectomy is preferred in patients with early-stage thymoma and no myasthenia gravis due to its shorter surgical time and fewer complications, extended thymectomy maintains its place in this field because simple thymectomy has worse results in terms of overall survival, 5-year survival, and recurrence-free survival (66). While minimally invasive methods (VATS/RATS) yield superior results in the postoperative period, patients with large size and invasion into surrounding tissues should also opt for open surgery (sternotomy) (59, 60).

Staudies have shown that while surgical results for stage IVA thymic tumors are acceptable, it is more accurate to make a multidisciplinary surgical decision due to the lack of data for stage 4B thymic tumors (21, 23, 24, 67). The surgical margin for advanced-stage thymic tumors is to provide R0 resection (21, 23, 24, 67). The stage IVA group, a heterogeneous group that includes both pleural and pericardial metastases, can resect pleural metastases, but pericardial metastases require careful evaluation (67, 68). Another difference in the stage IVA group is that recurrent and new pleural nodules were both staged the same way. However, thymic tumors that were found to be stage IV were more aggressive (68). Surgery for local control rather than adjuvant treatment is recommended for patients with recurrent pleural metastases (68).Re-operative surgery plays a critical role in the management of stage IV thymomas, particularly in cases where recurrence occurs after initial treatment. Stage IV thymomas, characterized by metastasis either to pleural or pericardial surfaces(IVA) or distant organs (stage IVB), often require multimodal treatment, including surgery, chemotherapy, and radiotherapy (67, 68). While complete surgical resection remains the cornerstone of treatment for early-stage thymomas, re-operative surgery can offer a significant survival benefit in advanced cases, especially for patients with localized recurrences or limited metastatic disease (68). Studies have shown that re-operative surgery can help achieve prolonged disease control and improve overall survival, particularly in patients who are candidates for resection of recurrent pleural nodules or other resectable metastases. However, the decision for re-operation must be individualized, taking into account the patient's overall condition, the extent of disease, and prior treatments. Combining surgery with other modalities like chemotherapy or radiotherapy can further enhance outcomes in this challenging patient population.

Cytoreductive Surgery Followed by Intraoperative Hyperthermic Chemotherapy (HITHOC) is a method applied after extended thymectomy in advanced stage thymomas with pleural spread, and its success in terms of local control has been demonstrated (69). In HITHOC, the intrathoracic temperature is raised to 42.5°C after surgery, and the procedure is carried out with a chemotherapeutic agent for about an hour (69). This method is used for both initial abdominal and pleural cancers (69). Research has demonstrated that this method enhances overall survival in cases of pleural recurrence and advanced-stage thymic epithelial tumors (69).

## 2 Conclusion

Although myasthenia gravis is an autoimmune disease, surgery has an important role in its treatment (31, 49). Extended thymectomy is the gold standard for surgical treatment of myasthenia gravis (24, 60, 63, 64). Removing the thymus and peripheral fatty tissue between the bilateral phrenic nerves prevents relapses (24, 60, 63, 64, 70).

Surgical treatment is important for early-stage thymomas (24, 60). Staging is of great importance in determining the best possible treatment (15, 20, 21). In this regard, it is recommended that the N component of staging should not be overlooked and it is recommended to perform lymph node dissection carefully exclusively in type B thymomas and thymic carcinomas (21–24).
